# Supplementary material for: Effect of dietary protein levels on the growth, enzyme activity, and immunological status of Culter mongolicus fingerlings
Source: PLoS One. 2022 Feb 4;17(2):e0263507. doi: 10.1371/journal.pone.0263507 (PMC8815975; doi:10.1371/journal.pone.0263507)
Supplement: Attachment — Submitted filename: Response to Reviewers.doc [file pone.0263507.s002.doc]

**Response to Reviewers**

**Manuscript ID:** PONE-D-21-25983

**Reviewer #1**: Dear authors

Your data are new and practical, the manuscript is well-written, and the amout of data should be enough, depending on the journals policies.

Based on these, i would recommend your results for publication.

**Response**: Dear reviewer, thanks for your review and approving. Best wishes for you!

**Reviewer #2**: The manuscript investigated the "Effect of dietary protein levels on the growth, enzyme activity, and immunological status of *Culter mongolicus* fingerlings." The manuscript is well, hence readable. The authors studied a species that appear to new in aquaculture. If it is a new species in aquaculture, this research very relevant as determining nutritional requirement in fish is an important step in the adoption of the fish species in aquaculture. However, before this manuscript is accepted in this prestigious journal minor revision is required.

**Response**: Thank you very much for your support to our work. We carefully revised our manuscript according to your valuable comments and suggestions.

Keywords: avoid using words in the title of the manuscript.

**Response**: Thank you, we changed keywords to: *Culter mongolicus*, protein requirement, physiological and biochemical indexes

Line 68: this sentence needs to be specific on what is being referred to: is it fish or something else.

**Response**: It is fish. And the original text was amended to: “It is widely accepted that the level of fish digestive enzyme activity is a useful comparative indicator of food utilization, digestive capacity, and growth performance of the host” (Lines 67-68).

Line 90: cite your earlier research for better reference.

**Response**: We added the earlier paper, marked as NO.50, it is in Chinese with English abstract.

Line 107, what is the basis of the protein level, any reference?

**Response**: *Culter mongolicus* is a carnivorous species, and according to protein requirement of other carnivorous fish, we set experimental diets (Lines 233-234).

It is not clear in the introduction why protein requirement is being investigated on this particular species. Is it a new species in aquaculture, or old aquaculture species but there is lack of information on its nutritional requirement.

**Response**: *Culter mongolicus* is one new aquaculture species, people feed them with other carnivorous fish’s feed now, and there is lack of information on its nutritional requirement.

Line 136: ‘’A third of the water was replaced at 19:00 daily. Was’nt this stressful to the fish if we have to take ethics in consideration. Was the rearing system not recirculating. Another issue is the stocking density, don’t the author think 70 fish for this experiment were too many? The use of too many fish for this experiment can be unethical. Explain why you used this number of fish per tank.

**Response**: The new water has been aerated for one day, before slowly flowing into cylinders, so the temperature was the same with old water. With the water changing was slowly, the stress was ignored. And the rearing system was not recirculating.

The *Culter mongolicus* fingerlings are small, according to pre-experimentation, 70 fish in one tank was not crowded, and their growth did not stunted.

Line 137: Although you mentioned that water parameters are presented as average, it is not clear. I suggest you use average/mean with standard deviation or standard error.

**Response**: We use air condition system for temperature control, and the new water has been aerated for one day, before slowly flowing into cylinders, so water parameters are presented as average.

Line 183 – Explain the regression formular; what each abbreviation stands for.

**Response**: weight-specific growth rate was estimated using broken-line regression analysis (y = L − U × (R − x), y means specific growth rate, x means protein content).

Figure 3: The y-axis label needs to be revised. This figure does not represent digestive enzymes but rather non-specific immune parameters/ biochemical parameters. And you may want to change the colour of lysozyme and MDA so that they are clearly distinct.

**Response**: Thank you for your suggestion, we revised Fig. 3 accordingly.
